# The effect of intravenous vitamin C on clinical outcomes in patients with sepsis or septic shock: A meta-analysis of randomized controlled trials

**DOI:** 10.3389/fnut.2022.964484

**Published:** 2022-07-28

**Authors:** Huiyan Zhu, Xiaoya Xu, Kai Zhang, Qiaoping Ye

**Affiliations:** ^1^Department of General Surgery, Lishui People’s Hospital, Lishui, China; ^2^Department of Critical Care Medicine, Second Affiliated Hospital, Zhejiang University School of Medicine, Hangzhou, China

**Keywords:** vitamin C, ascorbic acid, sepsis, septic shock, meta-analysis

## Abstract

**Objectives:**

Vitamin C deficiency is common among patients with sepsis and has been associated with poor clinical outcomes. Nevertheless, the effect of intravenous (IV) vitamin C for the treatment of sepsis remains controversial. The purpose of this meta-analysis was to evaluate the effect of IV vitamin C in patients with sepsis or septic shock.

**Methods:**

Electronic databases (PubMed, Embase, Scopus, and Cochrane Library) were searched from inception through May 25, 2022 for randomized controlled trials evaluating the effect of IV vitamin C treatment in patients with sepsis. The primary outcome was short-term mortality, and secondary outcomes including the duration of vasopressor, length of intensive care unit (ICU) stay, and Sequential Organ Failure Assessment (SOFA) score after vitamin C treatment. Subgroup analyses were performed based on the type of disease, dose and duration of IV vitamin C.

**Results:**

A total of 10 studies were included, with a total sample of 755 septic patients. The IV vitamin C was associated with a significant reduction in the short-term mortality (OR 0.51, 95% CI 0.37–0.69, *I*^2^ = 0%) and duration of vasopressor (MD −27.88, 95% CI −49.84 to −5.92, *I*^2^ = 95%). The length of ICU stay (MD −0.68, 95% CI −2.13 to 0.78, *I*^2^ = 74%) and SOFA score (MD −0.05, 95% CI −1.69 to 1.58, *I*^2^ = 86%) were not significantly different between two groups.

**Conclusion:**

In patients with sepsis or septic shock, the IV vitamin C reduced the short-term mortality rate and duration of vasopressor, with no effect on the length of ICU stay and SOFA score. Further trials are required to explore the optimal dosage and duration of IV vitamin C.

**Systematic Review Registration:**

https://inplasy.com/inplasy-2022-6-0013/, identifier INPLASY202260013.

## Introduction

Sepsis is a life-threatening syndrome associated with physiological, pathological, and biological abnormalities due to a dysregulated immune response to infections (1). Despite the advances in sepsis research improving the diagnosis and treatment (2), sepsis continues to be the most major cause of intensive care unit (ICU) admission and death (3, 4). Based on the Global Burden of Diseases 2017 estimates, there were 48.9 million incident sepsis cases worldwide, with nearly 11.0 million sepsis-related deaths, accounting for 19.7% of all global deaths (5). Even survivors are at high risk of developing functional limitations, cognitive impairment, and mental health problems, which significantly impair the quality of life (6).

It is well-established that patients with sepsis have decreased levels of vitamin C (also known as ascorbic acid), and this depletion has a dose-dependent association with increased organ dysfunction and mortality (7). The beneficial effects and associated mechanisms of vitamin C in sepsis including its anti-inflammatory and anti-oxidant properties (8, 9), acting as an enzymatic cofactor in the synthesis of vasopressin, cortisol, and catecholamine (10, 11), inhibiting the nitric oxide synthase and regulating the clearance of alveolar fluid (12, 13).

In recent years, multiple randomized controlled trials (RCTs) evaluating the effect of intravenous (IV) vitamin C with or without hydrocortisone and thiamine have been completed. Several recently published meta-analyses (14–20) assessed the combination of hydrocortisone, ascorbic acid, and thiamine (HAT) treatment in septic patients. The results indicated that the HAT treatment improved the Sequential Organ Failure Assessment (SOFA) score and reduced the duration of vasopressor, but was not associated with lower short-term mortality. However, the evidence-based medical evidence for using IV vitamin C as monotherapy in septic population are scarce. Therefore, this current study aimed to evaluate the effect of IV vitamin C alone on clinical outcomes among patients with sepsis or septic shock. Furthermore, we performed subgroup analyses to better understand the effectiveness of IV vitamin C in different populations and examined whether a dose-effect modified the treatment effect of vitamin C.

## Methods

### Data sources and study selection

We conducted our study on the basis of the updated PRISRMA statement (21) (checklist in Supplementary Material), and the study protocol was registered in International Platform of Registered Systematic Review and Meta-analysis Protocols (INPLASY 202260013). We systematically searched the PubMed, Embase, Scopus, and Cochrane Library for relevant studies in English from inception through May 25, 2022. The search used broad search terms containing “sepsis,” “septic shock,” “vitamin C,” “ascorbic acid,” and “randomized” (the comprehensive search strategies are listed in Supplementary Data Sheet 2).

### Eligibility criteria

The inclusion criteria were as follows: 1. Population: adult patients (≥18 years of age) with sepsis or septic shock. Sepsis was defined as reported by the original authors, septic shock was defined as sepsis with the need for vasopressor support; 2. Intervention: IV vitamin C as monotherapy; 3. Comparison: placebo, or no intervention; 4. Outcomes: the primary outcome was short-term mortality, including hospital mortality, and 28/30-day mortality. Secondary outcomes including the duration of vasopressor, length of ICU stay, and SOFA score after vitamin C treatment; 5. Design: RCT.

### Data extraction and quality assessment

Two authors (Huiyan Zhu, Qiaoping Ye) independently retrieved relevant studies, extracted characteristics of studies (first author, years of publication, population, intervention and control methods, vitamin C level) and predefined outcomes from included studies.

The Cochrane risk of bias tool (22) was utilized for assessing the methodological quality of including studies by two authors (Huiyan Zhu, Qiaoping Ye), any differences in opinion were resolved by a third adjudicator (Xiaoya Xu).

### Statistical synthesis and analysis

We computed the pooled odds ratio (OR) with 95% confidence interval (CI) for dichotomous outcomes, and mean difference (MD) with 95% CI for continuous outcomes. The heterogeneity was assessed by the Higgins inconsistency (*I*^2^) statistics (23). Substantial heterogeneity was identified when *I*^2^ value > 30% and a random-effects model was employed to perform the analysis, otherwise a fixed-effects model would be used. Publication bias was assessed by using the funnel plot and Egger’s regression test (24).

A prespecified subgroup analysis stratified by the types of disease (sepsis vs. septic shock), dose [high dose was set to a daily dose of ≥100 mg/kg or 10000 mg/day, according to the review of Patel et al. (25)], and duration [<5 days vs. ≥5 days, according to the study by Jung et al. (26)] of IV vitamin C treatment. Finally, a sensitivity analysis was conducted to explore the effect of individual study by consecutive exclusion of each study at one time.

## Results

### Study characteristics

**FIGURE 1:**
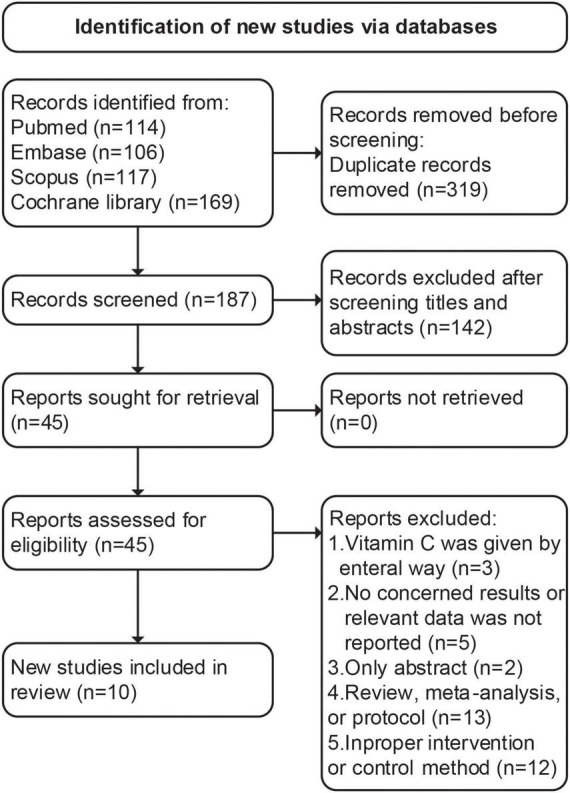
PRISMA 2020 flow diagram for the meta-analysis.

During the primary search, we identified 506 articles. Among them, 319 were duplicated articles, and 142 studies were excluded by screening the abstracts. During the evaluation of the full text, 35 studies were further removed with various reasons. Eventually, a total of ten RCTs (27–36) were included in our study (follow chart in Figure 1).

Table 1 presents the characteristics of included studies. A total of 755 patients were included in the analysis, whereof 384 patients received IV vitamin C and 371 patients received placebos or no intervention during the study period. The number of patients in each study ranged from a minimum of 20 up to 167. Seven trials (29–33, 35, 36) enrolled patients with sepsis or septic shock based on the Sepsis-3 criteria, three trials (27, 28, 34) diagnosed sepsis by the original investigators. According to whether the patients required vasopressor support, patients were categorized into sepsis (27–29, 35) and septic shock (30–34, 36) cohort. The dose and duration of IV vitamin C varied amongst included trials. Five trials administered low-dose vitamin C, four administered high-dose vitamin C, and one trial included one low-dose vitamin C cohort and one high-dose vitamin C cohort. In six trials (28, 29, 31–34), patients received IV vitamin C for 3–4 days, two trials (27, 35) administered vitamin C for 6–7 days, and the rest two trials (30, 36) administered vitamin C until ICU discharge. Four trials (28, 29, 31, 32) reported the pre-trial and post-trial plasma vitamin C level, the IV vitamin C treatment could increase the plasma vitamin C concentration. The serum level of vitamin C was significantly higher in intervention group compared with control group.

**TABLE 1:** Characteristics of studies included in the meta-analysis.

Study	Design	Sample size	Population	Interventions	Vitamin C status (μ mol/L)^a^	Outcomes
Rosengrave et al. (32)	Double-blind, randomized placebo-controlled trial	Total: 40 (Intervention: 20; Control: 20)	Adult patients with septic shock (Sepsis-3)	Intervention: vitamin C (100 mg/kg/day) for 4 days; Control: 5% dextrose	Baseline: Intervention: 10 (4, 13); Placebo: 8.2 (4.7, 11); 72 h: Intervention: 408 (227, 560); Placebo: 4.4 (3.1, 8.8)	Mortality (in-hospital, 30-day, 90-day), duration of vasopressor, ICU length of stay, SOFA score at 4-day
Wacker et al. (33)	Double-blind, randomized placebo-controlled trial	Total: 124 (Intervention: 60; Control: 64)	Adult patients within 24 h of onset of septic shock (Sepsis-3)	Intervention: vitamin C (6000 mg/day) for 4 days; Control: normal saline	NR	28-day mortality, duration of vasopressor, ICU length of stay
Mahmoodpoor et al. (31)	Double-blind, randomized placebo-controlled trial	Total: 80 (Intervention: 42; Control: 38)	Critically ill patients with severe pneumonia and SOFA ≥ 2 (Sepsis-3)	Intervention: vitamin C (60 mg/kg/day) for 4 days; Control: normal saline	Baseline: Intervention: 20.63 ± 12.74; Placebo: 22.77 ± 13.56; 72 h: Intervention: 79.20 ± 26.42; Placebo: 16.38 ± 10.33	In-hospital mortality, duration of vasopressor, SOFA score at 4-day, ICU length of stay
Zhang et al. (35)	Double-blind, randomized placebo-controlled trial	Total: 56 (Intervention: 27; Control: 29)	Critically ill patients with severe SARS-CoV-2-related pneumonia and SOFA ≥ 2 (Sepsis-3)	Intervention: vitamin C (24000 mg/day) for 7 days; Control: bacteriostatic water	NR	Mortality (28-day, in-hospital), SOFA score at 7-day, ICU length of stay, hospital length of stay
Lv et al. (30)	Randomized placebo-controlled trial	Total: 117 (Intervention: 61; Control: 56)	Adult ICU patients diagnosed with septic shock (Sepsis-3)	Intervention: vitamin C (3000 mg/day) until ICU discharge; Control: 5% dextrose	NR	28-day mortality, SOFA score at 3-day, ICU length of stay, duration of vasopressor
CITRIS-ALI trial (29)	Double-blind, randomized placebo-controlled trial	Total: 167 (Intervention: 84; Control: 83)	Adult patients diagnosed with sepsis (Sepsis-3) and developed ARDS	Intervention: vitamin C (200 mg/kg/day) for 4 days; Control: 5% dextrose	Baseline: Intervention: 22 (8, 39) Placebo: 22 (11, 37); 96 h: Intervention: 169 (87, 412); Placebo: 26 (9, 41)	28-day mortality, improvement in SOFA score, SOFA score at 4-day
Habib et al. (36)	Open-label, randomized controlled trial	Total: 100 (Intervention: 50; Control: 50)	Adult patients admitted to the critical care department with the diagnosis of septic shock (Sepsis-3)	Intervention: vitamin C (6000 mg/day) until ICU discharge; Control: conventional treatment	NR	In-hospital mortality, duration of vasopressor, ICU length of stay
Zabet et al. (34)	Double-blind, randomized placebo-controlled trial	Total: 28 (Intervention: 14; Control: 14)	Adult surgical critically ill patients with diagnosis of septic shock	Intervention: vitamin C (100 mg/kg/day) for 3 days; Control: 5% dextrose	NR	28-day mortality, duration of vasopressor, ICU length of stay
Fowler et al. (28)	Double-blind, randomized placebo-controlled trial	Total: 24 (Intervention: 16; Control: 8)	Adult patients with severe sepsis in the ICU	Intervention: vitamin C (50 mg/kg/day or 200 mg/kg/day) for 4 days; Control: 5% dextrose	Baseline: Low dose: 17 (14, 28) High dose: 17 (11, 50) Placebo: 20 (11, 45); 96 h: Low dose: 331 (110, 806) High dose: 3082 (1592, 5772) Placebo: 16 (7, 27)	28-day mortality
Ferrón-Celma et al. (27)	Double-blind, randomized placebo-controlled trial	Total: 20 (Intervention: 10; Control: 10)	Adult patients developed sepsis after abdominal surgery	Intervention: vitamin C (450 mg/day) for 6 days; Control: 5% dextrose	NR	In-hospital mortality

^a^The data represent median (IQR) or mean ± SD.

ICU, intensive care unit; mg, milligram; kg, kilogram; NR, not reported; SOFA, sequential organ failure assessment; ARDS, acute respiratory distress syndrome.

In addition, the duration of vasopressor, length of ICU stay, and SOFA score were expressed in the form of median and interquartile range in several trials. Thus, we used the methodology of Wan et al. (37) to convert these data into mean and standard deviation.

### Quality assessment

**FIGURE 2:**
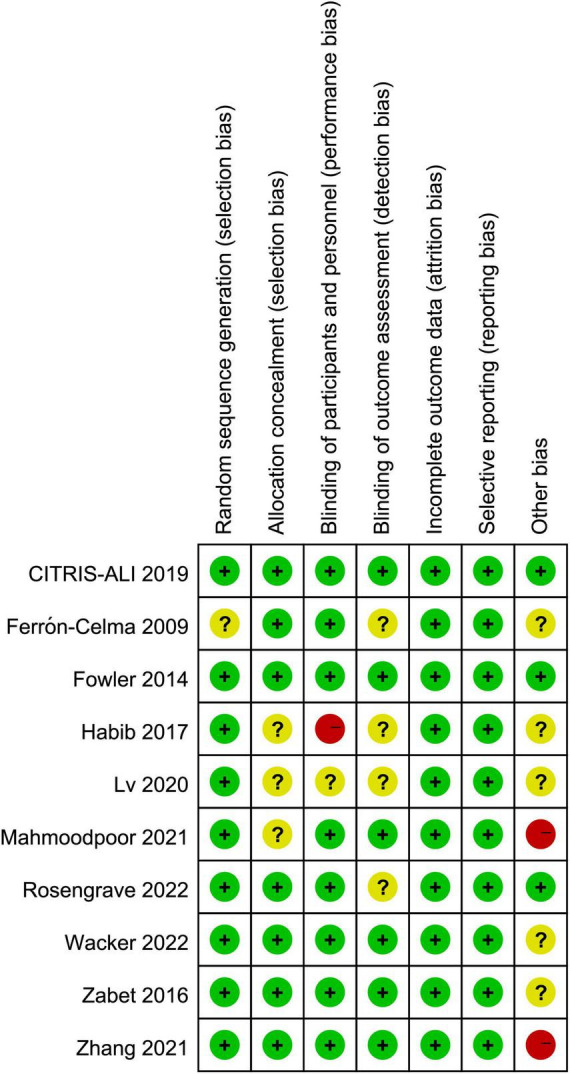
Assessment of quality by the Cochrane risk of bias tool.

The results of risk of bias assessment (Figure 2) showed that three studies were rated as high risk of bias: Habib et al. (36) used an open-label design, which carried the risk of bias; in the trial by Mahmoodpoor et al. (31), the SOFA score in intervention group was significantly higher than that in control group; Zhang et al. (35) enrolled critically ill patients with severe SARS-CoV-2-related pneumonia and SOFA score ≥ 2, which were different from other trials. Four trials (27, 30, 31, 36) did not provide the methods of random sequence generation or allocation concealment, four trials (27, 30, 32, 36) did not report the blinding method, which would either underestimate or overestimate the size of the effect. Moreover, six trials (27, 30, 32–34, 36) were rated as having an unclear risk of other bias since because the serum level of vitamin C of intervention and control groups were not provided.

In terms of publication bias, the funnel plot and Egger’s test showed that there was no significant risk of publication bias (Egger’s test, *P* > 0.05; Supplementary Data Sheet 1).

### Primary outcome

**FIGURE 3:**
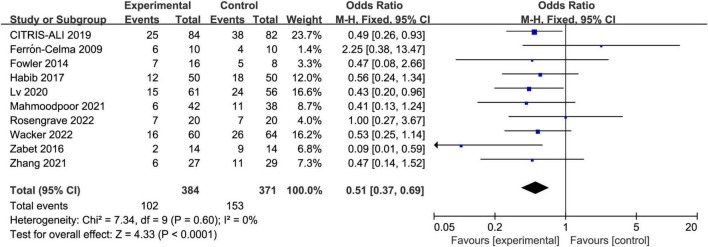
Forest plot showing the association between IV vitamin C and the risk of short-term mortality.

All included trials reported the short-term mortality with different definitions. Three trials reported in-hospital mortality, five trials reported 28-day mortality, two trials reported multiple results and we chose in-hospital mortality in the analysis. The incidence of short-term mortality in vitamin C group was lower than that in control group, 26.6% (102/384) vs. 41.2% (153/371), respectively. The pooled result indicated that IV vitamin C treatment was associated with a significant lower short-term mortality (OR 0.51, 95% CI 0.37 to 0.69, *I*^2^ = 0%, Figure 3).

### Secondary outcomes

**FIGURE 4:**
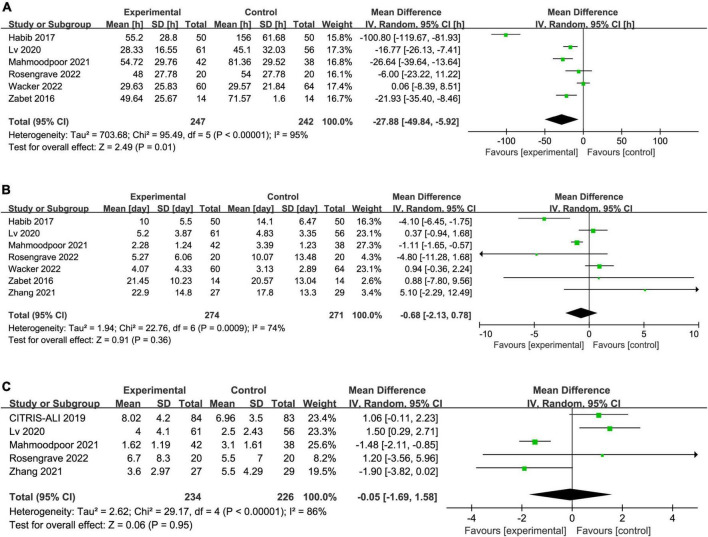
Forest plot showing the association between IV vitamin C and **(A)** duration of vasopressor, **(B)** length of ICU stay, **(C)** SOFA score.

Six trials reported the duration of vasopressor, seven reported the length of ICU stay, and five reported the SOFA score. The IV vitamin C treatment was associated with a reduction in the duration of vasopressor (MD −27.88, 95% CI −49.84 to −5.92, *I*^2^ = 95%, Figure 4A) among patients with septic shock. However, there was no significant difference in length of ICU stay (MD −0.68, 95% CI −2.13 to 0.78, *I*^2^ = 74%, Figure 4B) and SOFA score (MD −0.05, 95% CI −1.69 to 1.58, *I*^2^ = 86%, Figure 4C) between two groups. Notably, the results were greatly weakened by significant heterogeneity.

### Sensitivity and subgroup analysis

We assessed the effect of every single trial on the pooled result by omitting each study. Furthermore, the sensitivity analysis showed similar results to the overall analysis, indicating the good robustness (Supplementary Data Sheet 1).

We performed subgroup analyses to assess whether the types of disease, dose and duration of IV vitamin C treatment would affect the clinical outcomes. Four trials enrolled patients with sepsis (27–29, 35) and six enrolled patients with septic shock (30–34, 36). Five trials (27, 30, 31, 33, 36) administered low-dose vitamin C, four (29, 32, 34, 35) administered high-dose vitamin C, and one trial (28) included one low-dose vitamin C cohort and one high-dose vitamin C cohort. In six trials (28, 29, 31–34), patients received IV vitamin C for 3–4 days, two trials (27, 35) administered vitamin C for 6–7 days, and the rest two trials (30, 36) administered vitamin C until ICU discharge.

**FIGURE 5:**
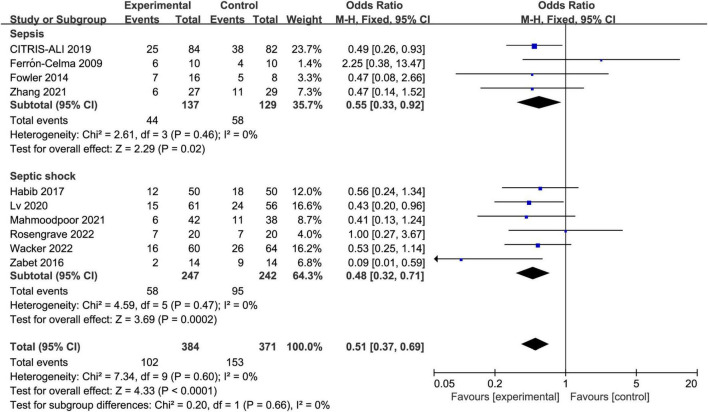
Forest plot showing the subgroup analysis of short-term mortality, patients with sepsis vs. patients with septic shock.

The IV vitamin C treatment was associated with a reduced mortality rate in both the patients with sepsis (OR 0.55, 95% CI 0.33–0.92, *I*^2^ = 0%, Figure 5) and septic shock (OR 0.48, 95% CI 0.32–0.71, *I*^2^ = 0%, Figure 5). Furthermore, the survival benefit was not associated with the dose or duration of IV vitamin C.

In terms of the duration of vasopressor, the high-does IV vitamin C was associated with reduction in the duration of vasopressor (MD −24.42, 95% CI −47.19 to −1.66, *I*^2^ = 95%), whereas the low-does subgroup found no difference (MD −14.10, 95% CI −29.32 to 1.13, *I*^2^ = 91%). Moreover, we did not observe significant difference in the duration of vasopressor between patients received IV vitamin C < 5 or ≥5 days.

In addition, the types of disease, dose and duration of IV vitamin C treatment did not have significant effects on the length of ICU and SOFA score (Supplementary Data Sheet 1). The results of subgroup analyses are shown in Table 2.

**TABLE 2:** Main findings and subgroup analysis.

Outcome	*N*	Result
**Mortality**	10	OR 0.51 (0.37, 0.69), *I*^2^ = 0% (*P* = 0.60)
Type of disease		
Sepsis	7	OR 0.55 (0.33, 0.92), *I*^2^ = 0% (*P* = 0.46)
Septic shock	3	OR 0.48 (0.32, 0.71), *I*^2^ = 0% (*P* = 0.47)
		Test for subgroup difference: *I*^2^ = 0%
Dose of vitamin C		
Low	6	OR 0.53 (0.35, 0.79), *I*^2^ = 0% (*P* = 0.68)
High	5	OR 0.48 (0.30, 0.77), *I*^2^ = 0% (*P* = 0.37)
		Test for subgroup difference: *I*^2^ = 0%
Duration of vitamin C		
<5 days	6	OR 0.48 (0.32, 0.71), *I*^2^ = 0% (*P* = 0.49)
≥5 days	4	OR 0.55 (0.34, 0.90), *I*^2^ = 0% (*P* = 0.42)
		Test for subgroup difference: *I*^2^ = 0%
**Duration of vasopressor**	6	MD −27.88, 95% CI −49.84 to −5.92, *I*^2^ = 95% (*P* < 0.00001)
Dose of vitamin C		
Low	3	MD −14.10, 95% CI −29.32 to 1.13, *I*^2^ = 91% (*P* < 0.00001)
High	5	MD −24.42, 95% CI −47.19 to −1.66, *I*^2^ = 95% (*P* < 0.00001)
		Test for subgroup difference: *I*^2^ = 0%
Duration of vitamin C		
<5 days	4	MD −13.37, 95% CI −27.42 to 0.68, *I*^2^ = 80% (*P* = 0.002)
≥5 days	2	MD −58.37, 95% CI −140.71 to 23.97, *I*^2^ = 98% (*P* < 0.00001)
		Test for subgroup difference: *I*^2^ = 10%
**Length of ICU stay**	7	MD −0.68, 95% CI −2.13 to 0.78, *I*^2^ = 74% (*P* = 0.0009)
Type of disease		
Sepsis	1	MD 5.10, 95% CI −2.29 to 12.49
Septic shock	6	MD −0.86, 95% CI −2.30 to 0.57, *I*^2^ = 75% (*P* = 0.001)
		Test for subgroup difference: *I*^2^ = 59%
Dose of vitamin C		
Low	3	MD −0.60, 95% CI −1.86 to 0.67, *I*^2^ = 56% (*P* = 0.11)
High	6	MD −0.98, 95% CI −4.14 to 2.18, *I*^2^ = 72% (*P* = 0.003)
		Test for subgroup difference: *I*^2^ = 0%
Duration of vitamin C		
<5 days	4	MD −0.45, 95% CI −2.24 to 1.34, *I*^2^ = 69% (*P* = 0.02)
≥5 days	3	MD −0.53, 95% CI −4.55 to 3.48, *I*^2^ = 84% (*P* = 0.002)
		Test for subgroup difference: *I*^2^ = 0%
**SOFA score**	5	MD −0.05, 95% CI −1.69 to 1.58, *I*^2^ = 86% (*P* < 0.00001)
Type of disease		
Sepsis	2	MD −0.32, 95% CI −3.21 to 2.58, *I*^2^ = 85% (*P* = 0.01)
Septic shock	3	MD 0.17, 95% CI −2.38 to 2.73, *I*^2^ = 90% (*P* < 0.0001)
		Test for subgroup difference: *I*^2^ = 0%
Dose of vitamin C		
Low	2	MD −0.04, 95% CI −2.96 to 2.88, *I*^2^ = 95% (*P* < 0.0001)
High	3	MD −0.05, 95% CI −2.36 to 2.27, *I*^2^ = 71% (*P* = 0.03)
		Test for subgroup difference: *I*^2^ = 0%
Duration of vitamin C		
<5 days	3	MD −0.05, 95% CI −2.26 to 2.16, *I*^2^ = 86% (*P* = 0.0006)
≥5 days	2	MD −0.11, 95% CI −3.44 to 3.21, *I*^2^ = 88% (*P* = 0.003)
		Test for subgroup difference: *I*^2^ = 0%

ICU, Intensive Care Unit; OR, Odds Ratio; MD, Mean Difference; CI, Confidence Interval; SOFA, Sequential Organ Failure Assessment.

## Discussion

In this meta-analysis, we included 10 RCTs with 755 patients to analyze the effect of IV vitamin C as monotherapy in patients with sepsis or septic shock. The preliminary analysis showed that the IV vitamin C treatment for patients with sepsis or septic shock was associated with a significant reduction in short-term mortality, but with no effect on the length of ICU stay and SOFA score. Meanwhile, the use of IV vitamin C treatment might reduce the duration of vasopressor for patients with septic shock. Furthermore, the dose and duration of vitamin C showed no significant effect on the clinical outcomes.

To our knowledge, this is the first comprehensive meta-analysis of RCTs to evaluate the effect of IV vitamin C as monotherapy in patients with sepsis or septic shock. Since recent meta-analyses (14–20) failed to find the association between HAT treatment and improved mortality among patients with sepsis, further research evaluating the HAT treatment in sepsis appears to be less necessary. In contrast, Patel et al. (25) performed the first meta-analysis to evaluate the role of IV vitamin C monotherapy in critically ill patients and revealed a significant treatment effect. On that basis, we hypothesized that IV vitamin C monotherapy still remain protective effect against sepsis, and further evaluated the effects of IV vitamin C in patients with sepsis or septic shock. The results of our meta-analysis are approximately consistent with the research by Patel et al. (25) that IV vitamin C treatment was associated with a significant lower short-term mortality, whereas with no difference in length of ICU stay and SOFA score.

The possible mechanisms of the benefit of IV vitamin C treatment in patients with sepsis or septic shock can be explained in several ways. First of all, serum levels of vitamin C decline rapidly among septic patients, confirming their critical involvement in a worsening prognosis (38, 39). The IV vitamin C treatment could restore the plasma vitamin C concentration. All the trials analyzed in our meta-analysis reported an increased serum level of vitamin C in intervention group.

Secondly, some of the physiological effects of vitamin C are of great significance to improve the prognosis of septic patients. Vitamin C is an important antioxidant of the body (40), supports the synthesis of vasopressin, cortisol, and catecholamine (10, 11), increases lymphocytic and neutrophilic activity while attenuating neutrophil necrosis (41, 42). Furthermore, vitamin C also regulates gene expression of pro-inflammatory and coagulation (39, 43), nuclear cellular responses to stress and hypoxia (44), and orchestrates the immune system and circulating cytokine homeostasis in pleotropic ways (42). Therefore, the vital role of vitamin C and its depletion in septic states justifies the use of IV vitamin C in patients with sepsis or septic shock.

However, as suggested by the negative results of some secondary outcomes in our meta-analysis, the beneficial effect of reducing SOFA score or length of ICU stay does not happen. Previous research has demonstrated that some patients might experience hypovitaminosis C as early as 48 h after discontinuation of vitamin C infusion, regardless of the dosing regimen (45). Given that most included trials limited IV vitamin C use to a maximum of 4 days, sustained therapy may be needed to obtain the favorable effects of vitamin C over time. In the CITRIS-ALI trial (29), patients in the 4-day IV vitamin C treatment group had lower 28-day mortality rate, but the survival curve parallel to that of placebo after cessation of vitamin C infusion. The subgroup analysis showed that septic patients received high-dose vitamin C had lower mortality rate and shorter duration of vasopressor, indicating the improvements in clinical outcomes might be dose dependent. Considering the higher dose or longer medication time of IV vitamin C may have produced different results (46), the most effective dose of IV vitamin C and duration of treatment as well as its effects on clinical outcomes also remains to be seen.

However, our study has several limitations. First of all, this meta-analysis was limited by the small sample size of included RCTs, the sample size was relatively small (number of participants < 100 per arm), which may introduce small-study effects and get larger beneficial treatment effects conclusion (47).

Secondly, since sepsis is a clinically common syndrome with high heterogeneity, the studied population represents a heterogeneous population. For example, some were surgical patients after major operation, some had severe pneumonia or respiratory failure. Similarly, the clinical characteristics of included studies were heterogeneous. The baseline of vitamin C level, dose and duration of IV vitamin C, as well as the disease severity of enrolled patients are varied across all the studies. Thus, the pooled estimates should be interpreted with caution since the significant heterogeneity.

Moreover, renal impairment is one of the important adverse effects when people receive high-dose IV vitamin C (48). Considering only a few articles reported the incidence of acute kidney injury as vitamin C related adverse event, there was not enough data to evaluate the incidence of acute kidney injury between vitamin C and control group.

Finally, the data for continuous variables were reported using the median, interquartile range, or range in several trials, which were calculated into mean and standard deviation. But there was a certain deviation from the real value that leading to bias into our results.

## Conclusion

Among patients with sepsis or septic shock, the IV vitamin C treatment was associated with significant reduction in short-term mortality and duration of vasopressor. Although the statistical heterogeneity considerably weakens the conclusions, the observed favorable effect of IV vitamin C on reducing short-term mortality and duration of vasopressor should be considered. However, we cannot draw a definitive conclusion from this current meta-analysis regarding the optimal dosage or duration of IV vitamin C treatment. Further studies evaluating the effect of different dose or duration of IV vitamin C in septic population are warranted.

## Data availability statement

The original contributions presented in this study are included in the article/Supplementary material, further inquiries can be directed to the corresponding author.

## Author contributions

HZ and QY conceived the idea, performed the analysis, and drafted the initial writing of this manuscript. XX contributed to the collection and interpretation of data. KZ helped to frame the idea of the study and provided technical support. QY contributed to the revision of this manuscript and to the final approval of the version to be published. All authors contributed to the article and approved the submitted version.
