# Supplementary material for: The effect of intravenous vitamin C on clinical outcomes in patients with sepsis or septic shock: A meta-analysis of randomized controlled trials
Source: Front Nutr. 2022 Jul 28;9:964484. doi: 10.3389/fnut.2022.964484 (PMC9366349; doi:10.3389/fnut.2022.964484)
Supplement: Supplementary file 1 [file Data_Sheet_1.pdf]

Supplementary Material: Publication bias assessment by funnel plot and Egger's test, sensitivity analyses, subgroup analyses.

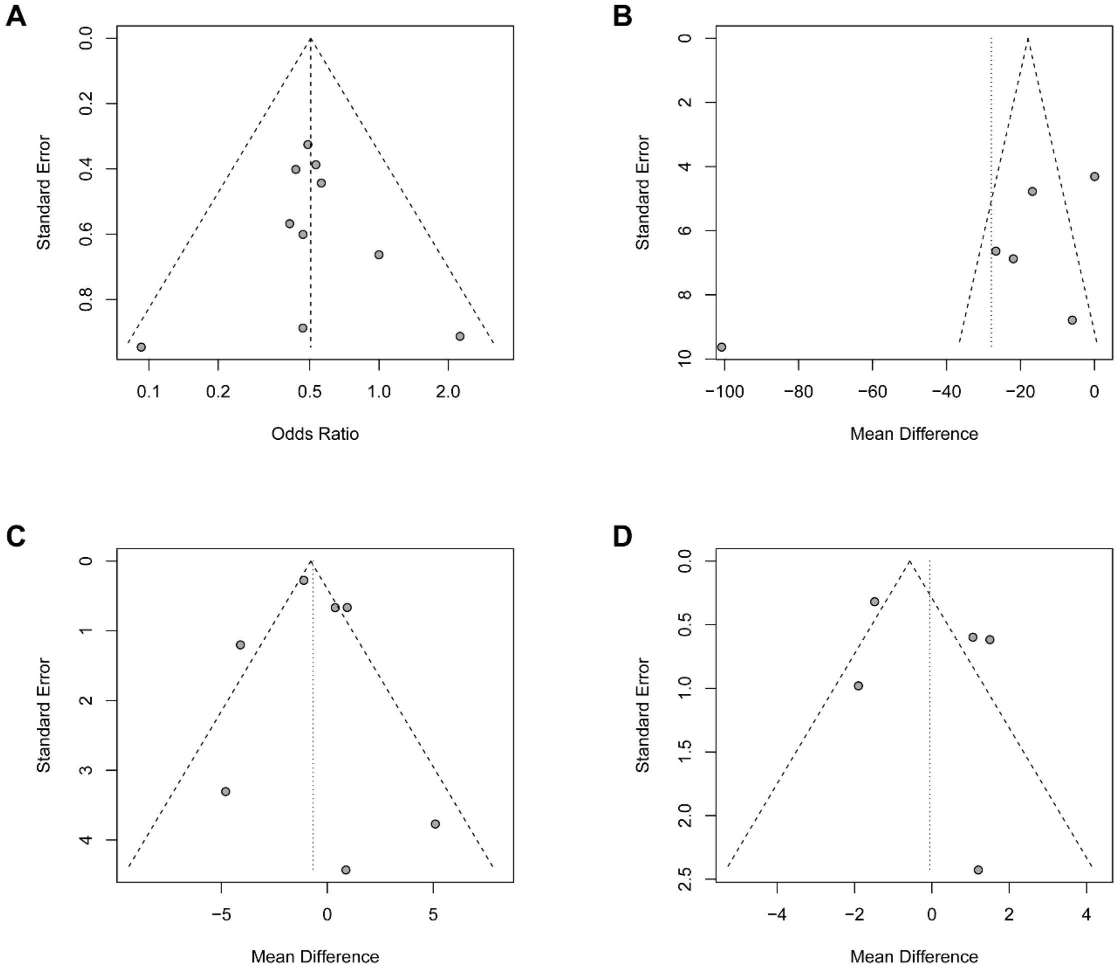

Figure 1: Funnel plot for (A) short-term mortality, Egger's test P=0.9116; (B) duration of vasopressor, Egger's test P=0.1241; (C) length of ICU stay, Egger's test P=0.7911; (D) SOFA score, Egger's test P=0.4663

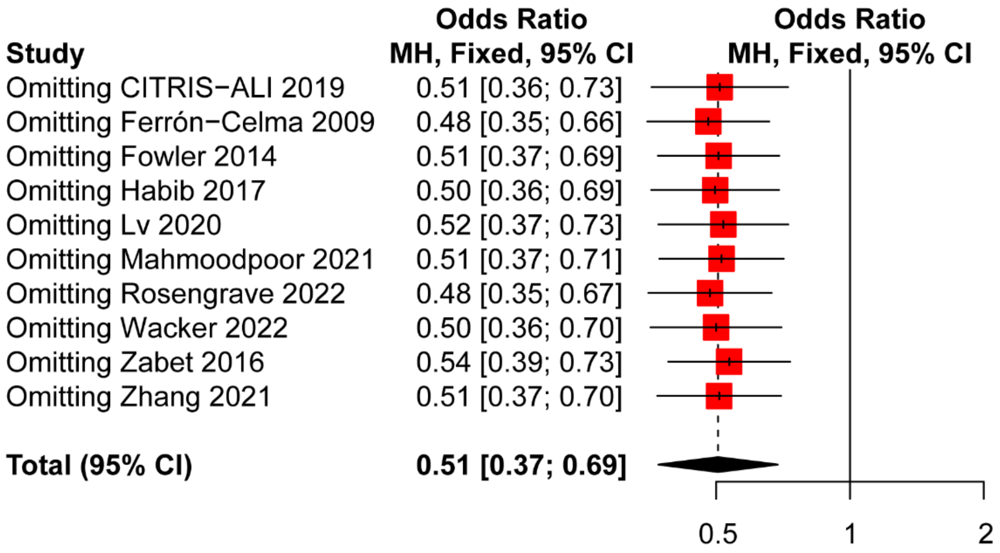

Figure 2: Sensitivity analysis for short-term mortality

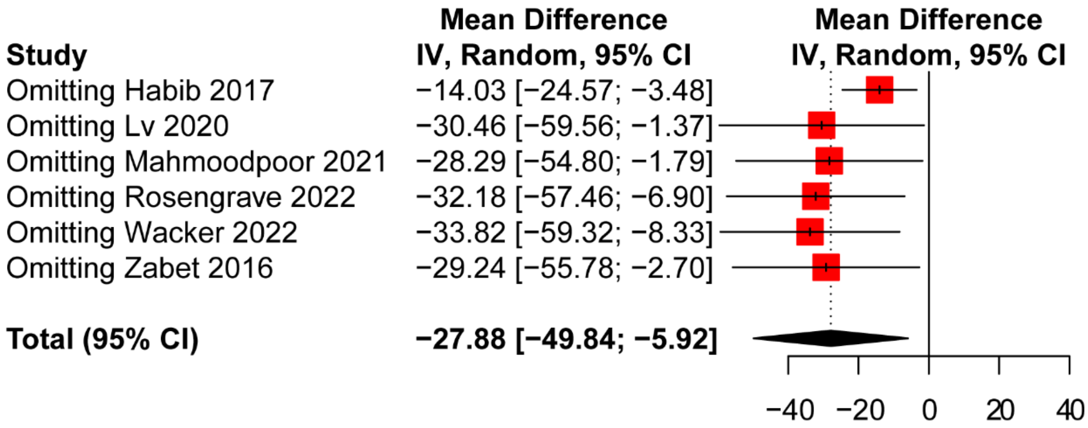

Figure 3: Sensitivity analysis for duration of vasopressor

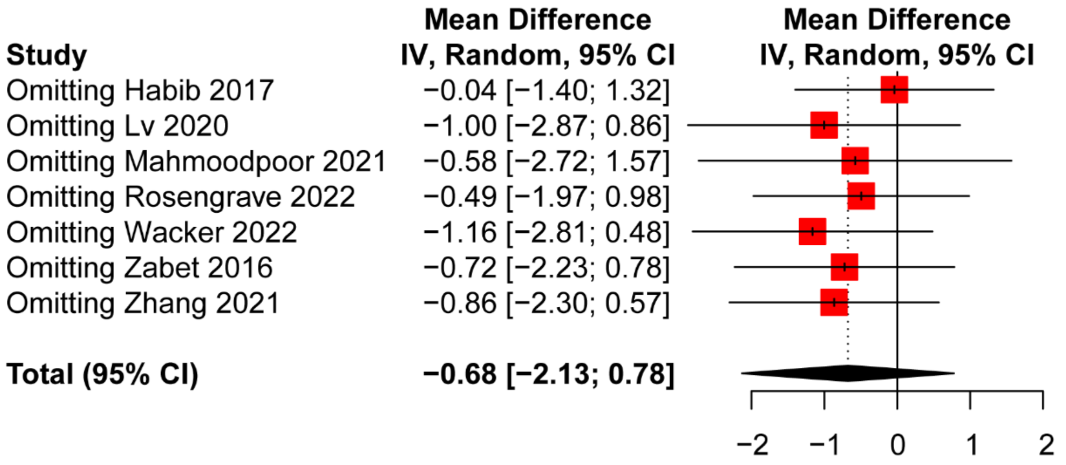

Figure 4: Sensitivity analysis for length of ICU stay

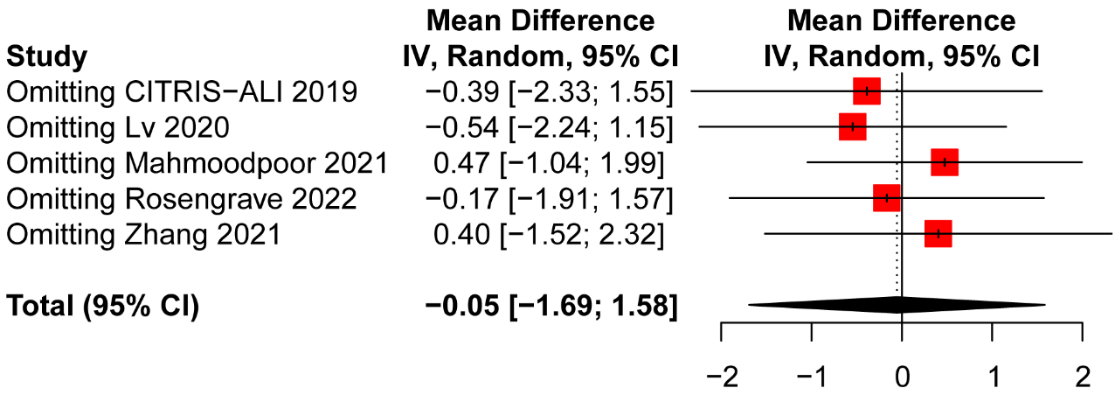

Figure 5: Sensitivity analysis for SOFA score

Study or Subgroup	Experimental Events	Experimental Total	Control Events	Control Total	Weight	Odds Ratio M-H, Fixed, 95% CI
**Low dose**						
Ferrón-Celma 2009	6	10	4	10	1.4%	2.25 [0.38, 13.47]
Fowler 2014 Cohort 1	3	8	5	8	2.7%	0.36 [0.05, 2.73]
Habib 2017	12	50	18	50	11.8%	0.56 [0.24, 1.34]
Lv 2020	15	61	24	56	16.3%	0.43 [0.20, 0.96]
Mahmoodpoor 2021	6	42	11	38	8.6%	0.41 [0.13, 1.24]
Wacker 2022	16	60	26	64	16.0%	0.53 [0.25, 1.14]
**Subtotal (95% CI)**		**231**		**226**	**56.7%**	**0.53 [0.35, 0.79]**
Total events	58		88			
Heterogeneity: Chi² = 3.11, df = 5 (P = 0.68); I² = 0%						
Test for overall effect: Z = 3.13 (P = 0.002)						
**High dose**						
CITRIS-ALI 2019	25	84	38	82	23.4%	0.49 [0.26, 0.93]
Fowler 2014 Cohort 2	4	8	5	8	2.2%	0.60 [0.08, 4.40]
Rosengrave 2022	7	20	7	20	3.9%	1.00 [0.27, 3.67]
Zabet 2016	2	14	9	14	6.7%	0.09 [0.01, 0.59]
Zhang 2021	6	27	11	29	7.1%	0.47 [0.14, 1.52]
**Subtotal (95% CI)**		**153**		**153**	**43.3%**	**0.48 [0.30, 0.77]**
Total events	44		70			
Heterogeneity: Chi² = 4.31, df = 4 (P = 0.37); I² = 7%						
Test for overall effect: Z = 3.07 (P = 0.002)						
**Total (95% CI)**		**384**		**379**	**100.0%**	**0.50 [0.37, 0.69]**
Total events	102		158			
Heterogeneity: Chi² = 7.47, df = 10 (P = 0.68); I² = 0%						
Test for overall effect: Z = 4.37 (P < 0.0001)						
Test for subgroup differences: Chi² = 0.09, df = 1 (P = 0.76), I² = 0%						

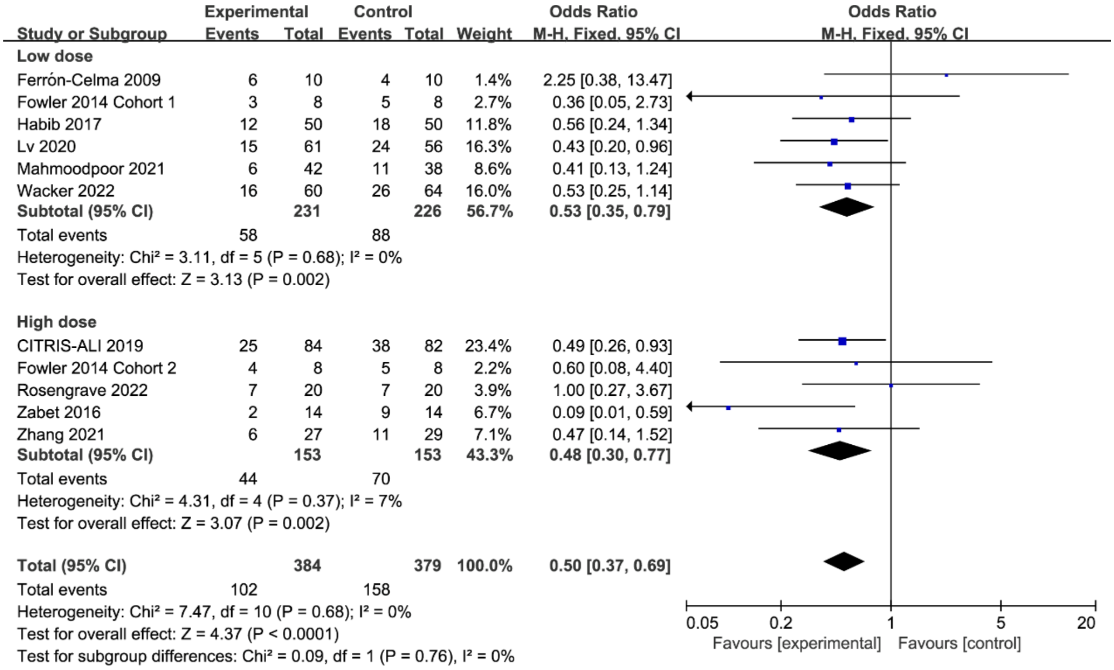

Figure 6: Subgroup analysis for short-term mortality, low dose versus high dose

Study or Subgroup	Experimental Events	Experimental Total	Control Events	Control Total	Weight	Odds Ratio M-H, Fixed, 95% CI
**<5 days**						
CITRIS-ALI 2019	25	84	38	82	23.7%	0.49 [0.26, 0.93]
Fowler 2014	7	16	5	8	3.3%	0.47 [0.08, 2.66]
Mahmoodpoor 2021	6	42	11	38	8.7%	0.41 [0.13, 1.24]
Rosengrave 2022	7	20	7	20	4.0%	1.00 [0.27, 3.67]
Wacker 2022	16	60	26	64	16.2%	0.53 [0.25, 1.14]
Zabet 2016	2	14	9	14	6.8%	0.09 [0.01, 0.59]
**Subtotal (95% CI)**		**236**		**226**	**62.7%**	**0.48 [0.32, 0.71]**
Total events	63		96			
Heterogeneity: Chi² = 4.41, df = 5 (P = 0.49); I² = 0%						
Test for overall effect: Z = 3.65 (P = 0.0003)						
**⩾5 days**						
Ferrón-Celma 2009	6	10	4	10	1.4%	2.25 [0.38, 13.47]
Habib 2017	12	50	18	50	12.0%	0.56 [0.24, 1.34]
Lv 2020	15	61	24	56	16.6%	0.43 [0.20, 0.96]
Zhang 2021	6	27	11	29	7.3%	0.47 [0.14, 1.52]
**Subtotal (95% CI)**		**148**		**145**	**37.3%**	**0.55 [0.34, 0.90]**
Total events	39		57			
Heterogeneity: Chi² = 2.80, df = 3 (P = 0.42); I² = 0%						
Test for overall effect: Z = 2.36 (P = 0.02)						
**Total (95% CI)**		**384**		**371**	**100.0%**	**0.51 [0.37, 0.69]**
Total events	102		153			
Heterogeneity: Chi² = 7.34, df = 9 (P = 0.60); I² = 0%						
Test for overall effect: Z = 4.33 (P < 0.0001)						
Test for subgroup differences: Chi² = 0.19, df = 1 (P = 0.66), I² = 0%						

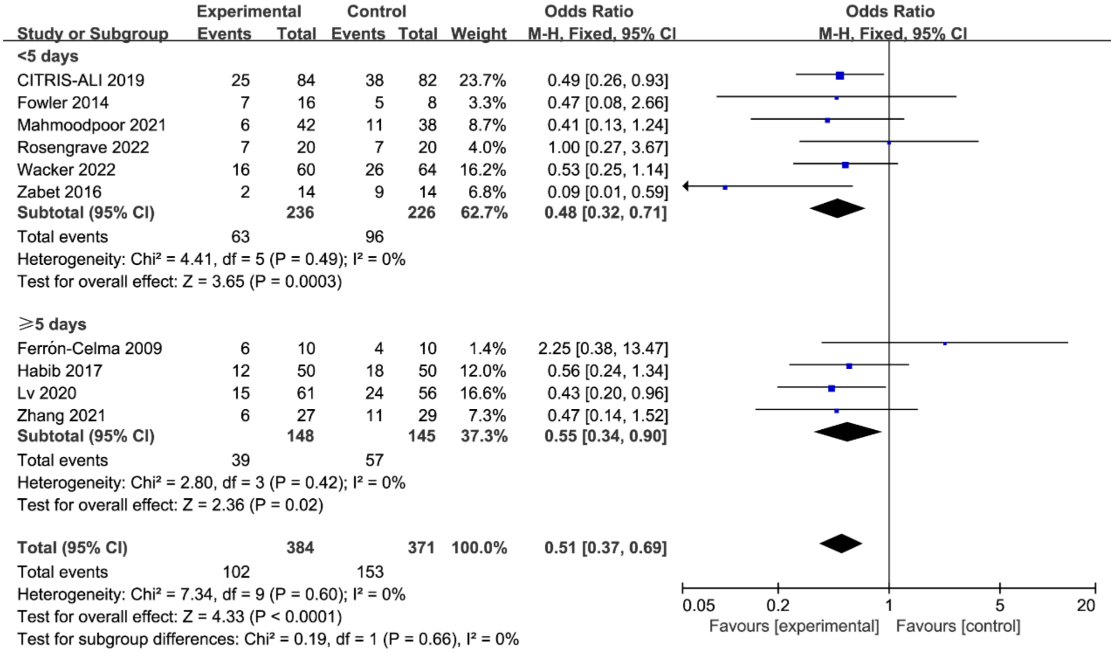

Figure 7: Subgroup analysis for short-term mortality, <5 days versus ⩾5 days

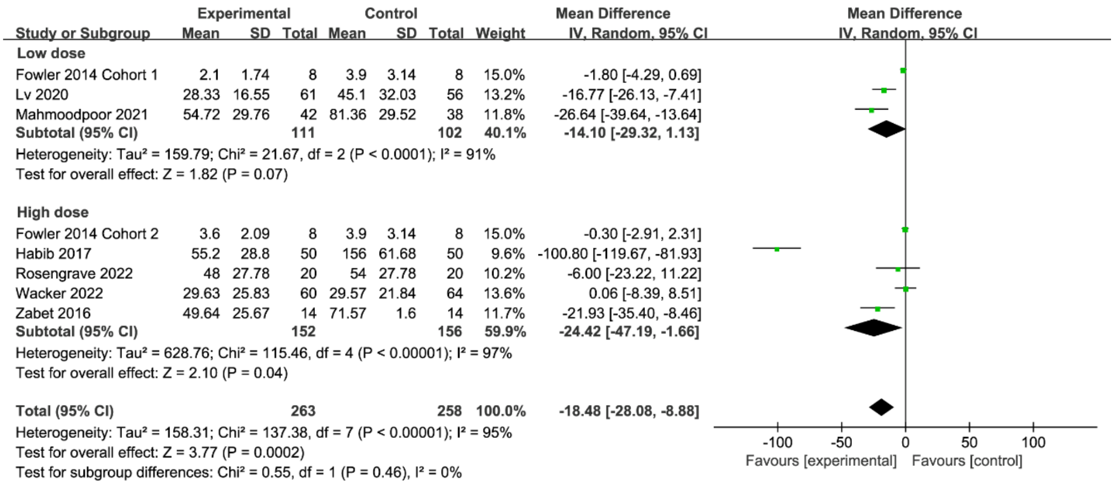

Figure 8: Subgroup analysis for duration of vasopressor, low dose versus high dose

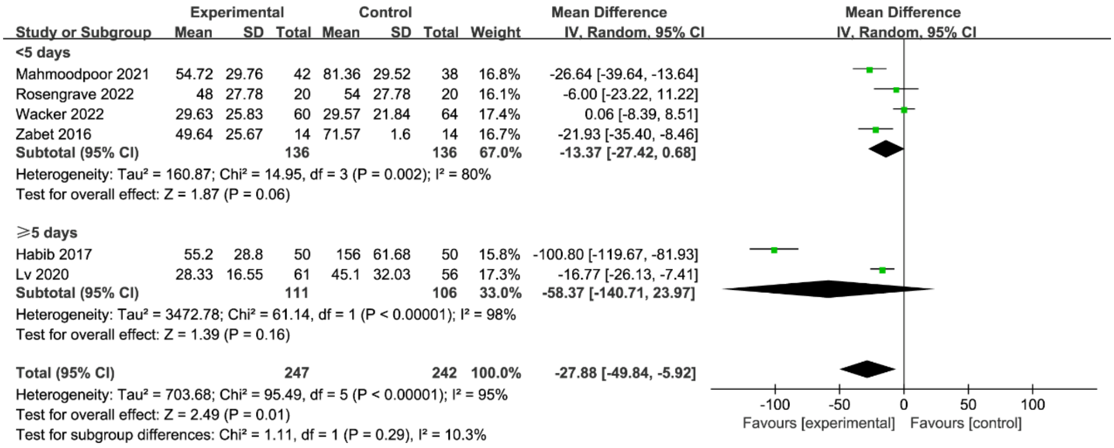

Figure 9: Subgroup analysis for duration of vasopressor, <5 days versus ⩾5 days

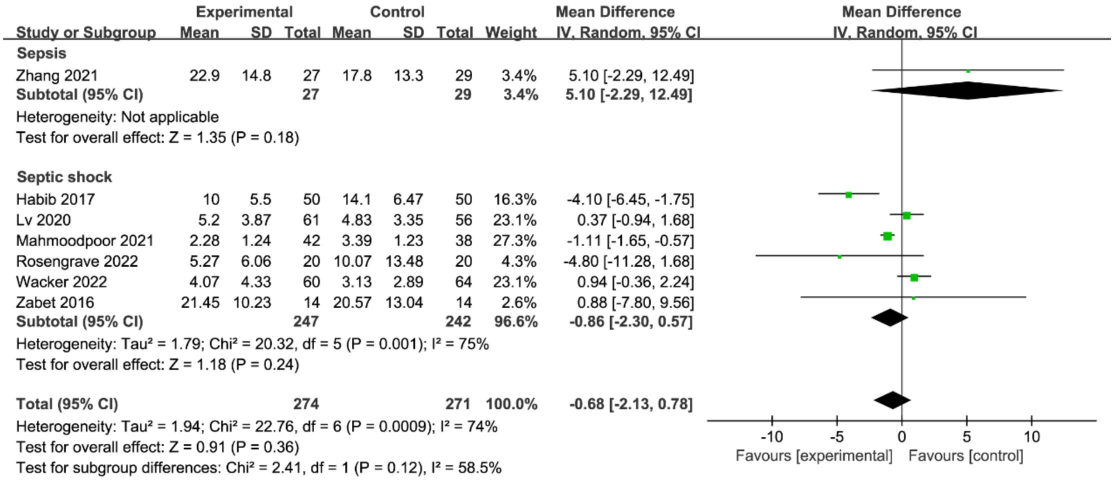

Figure 10: Subgroup analysis for length of ICU stay, patients with sepsis versus septic shock

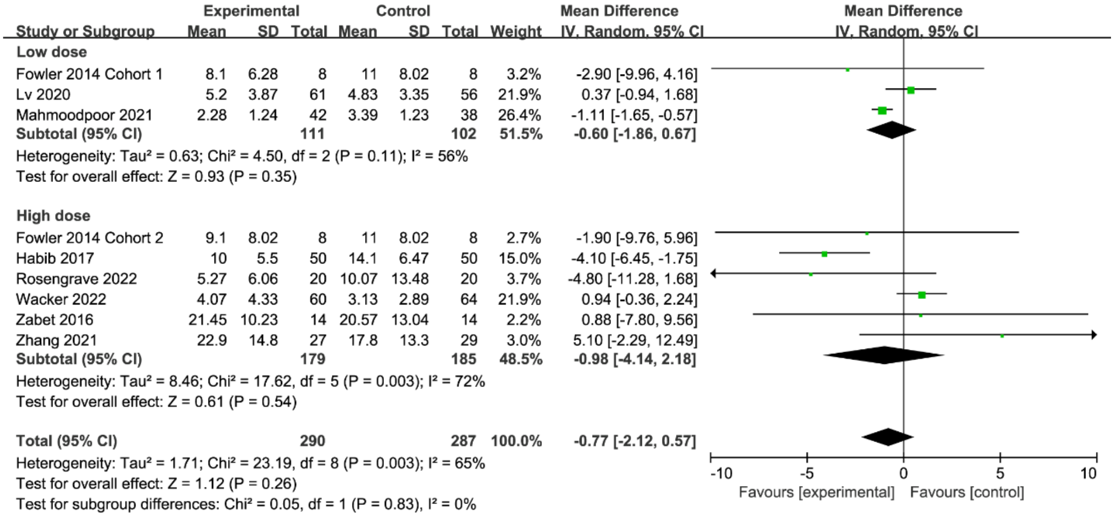

Figure 11: Subgroup analysis for length of ICU stay, low dose versus high dose

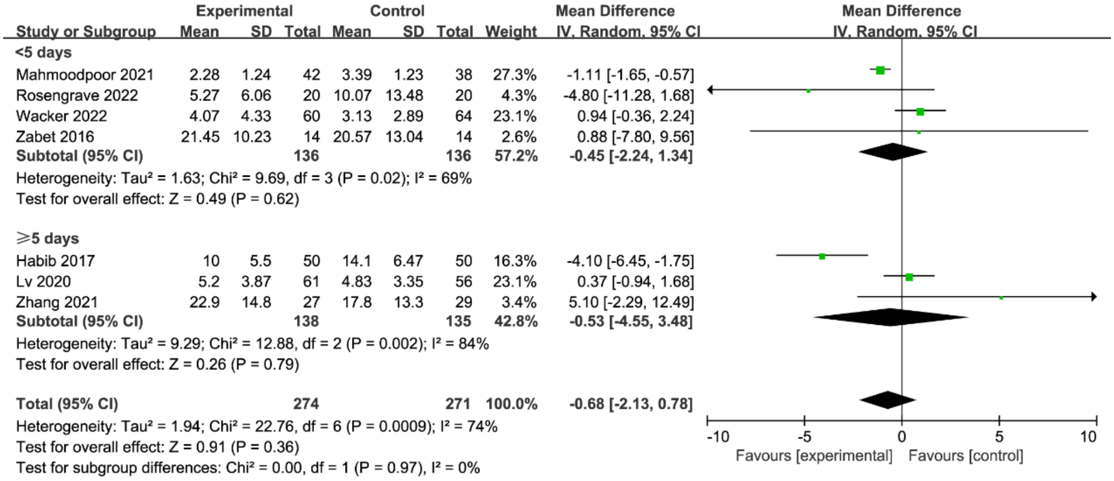

Figure 12: Subgroup analysis for length of ICU stay, <5 days versus ⩾5 days

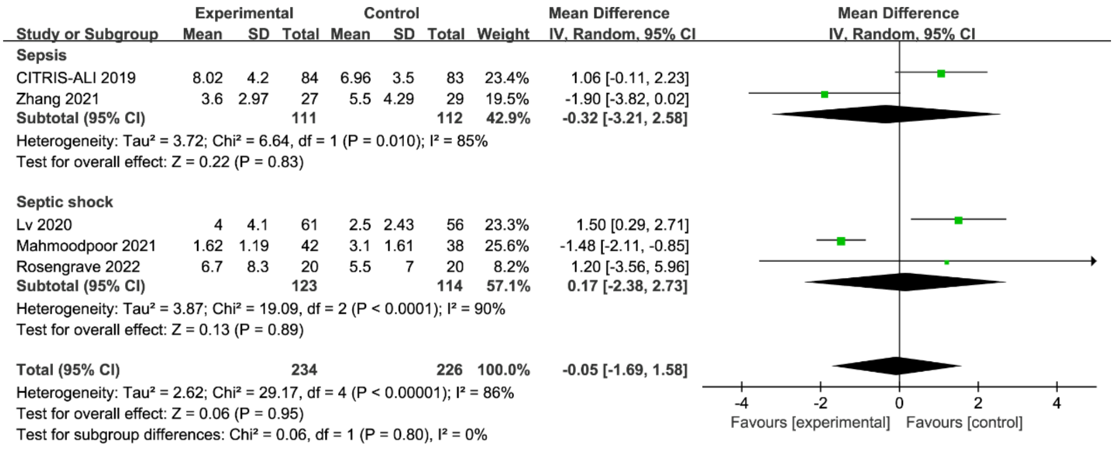

Figure 13: Subgroup analysis for SOFA score, patients with sepsis versus septic shock

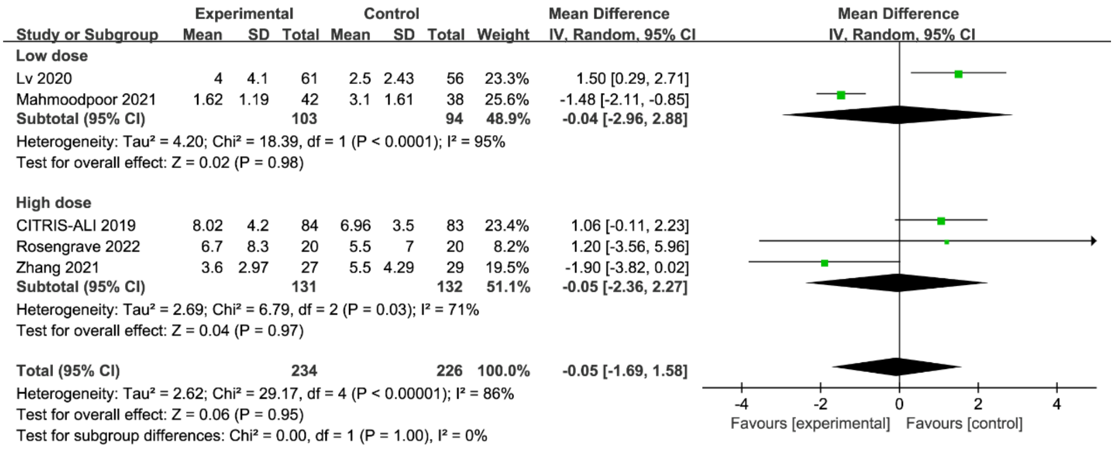

Figure 14: Subgroup analysis for SOFA score, low dose versus high dose

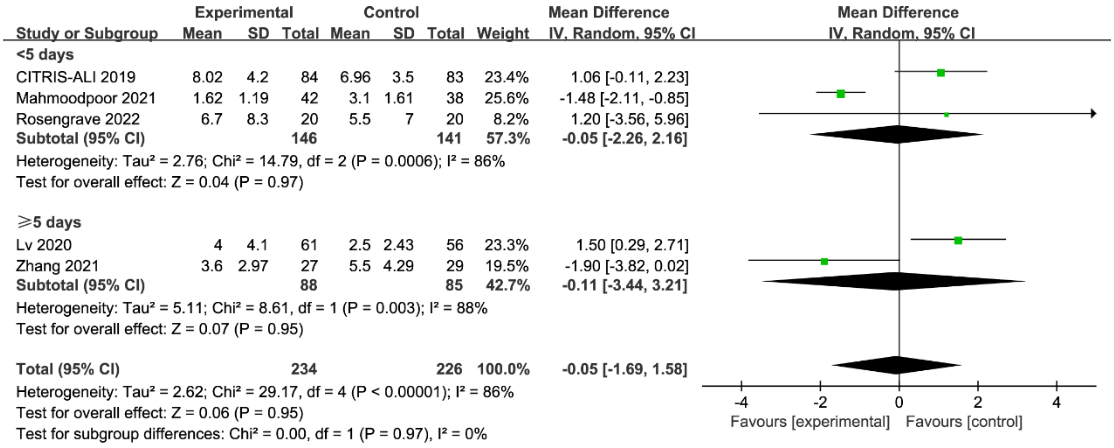

Figure 15: Subgroup analysis for SOFA score, <5 days versus ⩾5 days
