# Supplementary material for: The effect of intravenous vitamin C on clinical outcomes in patients with sepsis or septic shock: A meta-analysis of randomized controlled trials
Source: Front Nutr. 2022 Jul 28;9:964484. doi: 10.3389/fnut.2022.964484 (PMC9366349; doi:10.3389/fnut.2022.964484)
Supplement: Supplementary file 2 [file Data_Sheet_2.pdf]

Supplementary Material: Searching strategies

Pubmed 114

#1 sepsis [MeSH Terms] OR sepsis [Title/Abstract] OR septic [Title/Abstract]
#2 vitamin c [Title/Abstract] OR Ascorbic Acid [Title/Abstract] OR Ascorbic Acid [MeSH Terms]
#3 randomized controlled trial [MeSH Terms] OR randomised [Title/Abstract] OR randomized [Title/Abstract]
#1 AND #2 AND #3

Embase 106

#1 ‘sepsis’:ti,ab,kw OR ‘septic’:ti,ab,kw OR ' sepsis '/exp OR ' septic '/exp
#2 vitamin c:ti,ab,kw OR ' Ascorbic Acid '/exp
#3 'randomized controlled trial'/de OR 'randomized controlled trial'/exp
#1 AND #2 AND #3

Scopus 117

#1 TITLE-ABS-KEY (sepsis) OR TITLE-ABS-KEY (septic)
#2 TITLE-ABS-KEY (vitamin c) OR TITLE-ABS-KEY (Ascorbic Acid)
#3 TITLE-ABS-KEY (randomized) OR TITLE-ABS-KEY (random) OR TITLE-ABS-KEY (randomised)
#1 AND #2 AND #3

Cochrane Library 169

#1 (sepsis):ti,ab,kw OR (septic):ti,ab,kw
#2 (vitamin c):ti,ab,kw OR (Ascorbic Acid):ti,ab,kw
#3 (randomized):ti,ab,kw OR (randomised):ti,ab,kw OR (random):ti,ab,kw
#1 AND #2 AND #3
